# Brain-specific heterozygous loss-of-function of ATP2A2, endoplasmic reticulum Ca^2+^ pump responsible for Darier’s disease, causes behavioral abnormalities and a hyper-dopaminergic state

**DOI:** 10.1093/hmg/ddab137

**Published:** 2021-06-08

**Authors:** Kazuo Nakajima, Mizuho Ishiwata, Adam Z Weitemier, Hirotaka Shoji, Hiromu Monai, Hiroyuki Miyamoto, Kazuhiro Yamakawa, Tsuyoshi Miyakawa, Thomas J McHugh, Tadafumi Kato

**Affiliations:** Laboratory for Molecular Dynamics of Mental Disorders, RIKEN Center for Brain Science, Saitama 351-0198, Japan; Laboratory for Molecular Dynamics of Mental Disorders, RIKEN Center for Brain Science, Saitama 351-0198, Japan; Laboratory for Molecular Dynamics of Mental Disorders, RIKEN Center for Brain Science, Saitama 351-0198, Japan; Laboratory for Circuit and Behavioral Physiology, RIKEN Center for Brain Science, Saitama, Japan; Division of Systems Medical Science, Institute for Comprehensive Medical Science, Fujita Health University, Toyoake, Aichi 470-1192, Japan; Laboratory for Neuron-Glia Circuitry, RIKEN Center for Brain Science, Saitama, Japan; Faculty of Core Research Natural Science Division, Ochanomizu University, Tokyo 112-8610, Japan; Laboratory for Neurogenetics, RIKEN Center for Brain Science, Saitama, Japan; Laboratory for Neurogenetics, RIKEN Center for Brain Science, Saitama, Japan; Department of Neurodevelopmental Disorder Genetics, Nagoya City University Graduate School of Medical Sciences, Institute of Brain Science, Nagoya, Aichi 467-8601, Japan; Division of Systems Medical Science, Institute for Comprehensive Medical Science, Fujita Health University, Toyoake, Aichi 470-1192, Japan; Laboratory for Circuit and Behavioral Physiology, RIKEN Center for Brain Science, Saitama, Japan; Laboratory for Molecular Dynamics of Mental Disorders, RIKEN Center for Brain Science, Saitama 351-0198, Japan; Department of Psychiatry and Behavioral Science, Juntendo University Graduate School of Medicine, Tokyo 113-8421, Japan

## Abstract

A report of a family of Darier’s disease with mood disorders drew attention when the causative gene was identified as *ATP2A2* (or *SERCA2*), which encodes a Ca^2+^ pump on the endoplasmic reticulum (ER) membrane and is important for intracellular Ca^2+^ signaling. Recently, it was found that loss-of-function mutations of *ATP2A2* confer a risk of neuropsychiatric disorders including depression, bipolar disorder and schizophrenia. In addition, a genome-wide association study found an association between *ATP2A2* and schizophrenia. However, the mechanism of how *ATP2A2* contributes to vulnerability to these mental disorders is unknown. Here, we analyzed *Atp2a2* heterozygous brain-specific conditional knockout (hetero cKO) mice. The ER membranes prepared from the hetero cKO mouse brain showed decreased Ca^2+^ uptake activity. In *Atp2a2* heterozygous neurons, decays of cytosolic Ca^2+^ level were slower than control neurons after depolarization. The hetero cKO mice showed altered behavioral responses to novel environments and impairments in fear memory, suggestive of enhanced dopamine signaling. *In vivo* dialysis demonstrated that extracellular dopamine levels in the NAc were indeed higher in the hetero cKO mice. These results altogether indicate that the haploinsufficiency of *Atp2a2* in the brain causes prolonged cytosolic Ca^2+^ transients, which possibly results in enhanced dopamine signaling, a common feature of mood disorders and schizophrenia. These findings elucidate how *ATP2A2* mutations causing a dermatological disease may exert their pleiotropic effects on the brain and confer a risk for mental disorders.

## Introduction

Bipolar disorder is a mental disorder that shares many clinical features and pathophysiological basis with schizophrenia (1). Calcium signaling abnormalities have been proposed as the most likely hypothesis for the pathophysiology of bipolar disorder (2), which has been supported by genome wide association studies (GWAS) that identified *CACNA1C* encoding L-type calcium channel (3). However, the effect of the single nucleotide polymorphisms on bipolar disorder is modest with an odds ratio less than 1.2, and modeling in animals is still difficult. In such a situation, animal models of Mendelian diseases that show a bipolar disorder phenotype are therefore a promising strategy. Among such Mendelian diseases, a report of a family of Darier’s disease, an autosomal dominantly inherited skin disorder, in which major affective disorders including bipolar disorder co-segregated (4), is of particular interest because the causative gene was found to be *ATP2A2*, which encodes SERCA2, a Ca^2+^ pump on the endoplasmic reticulum (ER) (5–8). A recently released dataset from the Bipolar Exome (BipEx) sequencing project (https://bipex.broadinstitute.org/) showed an association between damaging missense mutations of *ATP2A2* and bipolar disorder (*P* = 0.005 with odds ratio 10). Similarly, a GWAS of schizophrenia also showed an association with *ATP2A2* and schizophrenia (9).

Patients with Darier’s disease have a higher prevalence rate of mood disorder (50%) including depression and bipolar disorder (10). Recent evidence has shown that loss of function (LOF) mutations of *ATP2A2* confer an increased risk for psychoses including bipolar disorder and schizophrenia (11). The C560R mutation in *ATP2A2* in the initial family was shown to drastically decrease expression levels of the ATP2A2 protein (11,12). These findings suggest that heterozygous KO mice of *Atp2a2* would be useful to study the role of calcium signaling in bipolar disorder and schizophrenia. However, conventional *Atp2a2* heterozygous KO mice showed serious somatic abnormalities (13), which has confounded behavioral analyses. Thus, a brain-specific heterozygous KO mouse of *Atp2a2* would be more useful to study bipolar disorder and schizophrenia.

Ca^2+^ uptake activity into the ER via ATP2A2 plays an essential role supporting cellular Ca^2+^ homeostasis by maintaining a low cytosolic Ca^2+^ concentration (14–16). As the ER is a widely distributed organelle in neurons including the cell body, dendrites, and axon terminals (17–19), ATP2A2 is thought to play an important role in a variety of neuronal functions. However, the *in vivo* significance of ATP2A2 in neurons and in the brain has not been studied as extensively compared to other cell types or organs (20). Considering the essential roles of ATP2A2 in Ca^2+^ signaling, mood disorders and schizophrenia in Darier’s disease patients, which are supposed to result from a pleiotropic effect of the LOF mutations in *ATP2A2*, a study of brain-specific KO mice for *Atp2a2* would be useful to clarify the mechanisms. In this study, we generated and analyzed brain-specific KO mice for *Atp2a2*. ATP2A2 was shown to be essential for neuronal functions including Ca^2+^ homeostasis, and the brain-specific heterozygous KO mice showed characteristic behavioral phenotypes. Enhanced dopamine (DA) neurotransmission was also detected in the nucleus accumbens (NAc). These findings may explain how *ATP2A2* confers risk for psychoses including bipolar disorder and schizophrenia.

## Results

### Generation of *Atp2a2* brain-specific heterozygous KO mice

**Figure 1:**
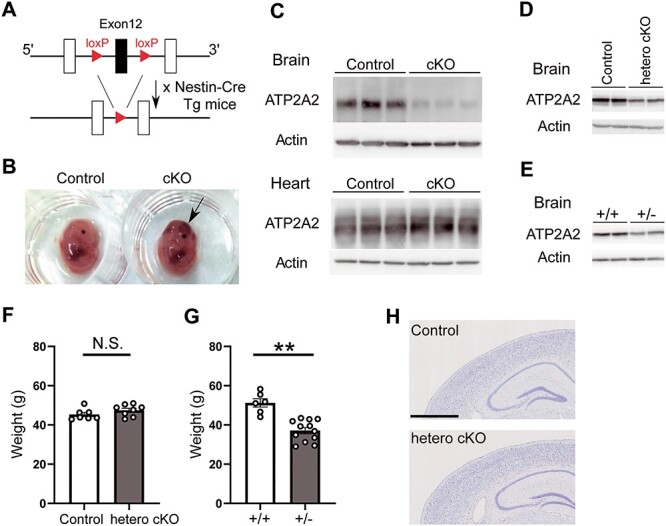
Generation of *Atp2a2* brain-specific conditional knockout (cKO) mice. (**A**) Schematic diagram of the targeting strategy. (**B**) The appearance of the *Atp2a2* brain-specific homozygous KO mice. Arrow, hemorrhage. (**C**) Western blotting using tissue lysates (E14). (**D**, **E**) Western blotting using brain lysates of the *Atp2a2* brain-specific heterozygous knockout (hetero cKO) mice (D) and conventional heterozygotes (E), male mice of 3–4 months of age were used. (**F**) Body weight of the control and hetero cKO male mice (*n* = 7, control; *n* = 8, hetero cKO). (**G**) Body weight of the wild-type and conventional heterozygous male mice (*n* = 6, +/+; *n* = 12, +/−). (**H**) Histology of the coronal sections of the brain in the control and hetero cKO mice. Scale bar, 1000 μm. ^*^^*^, *P* < 0.01.

We generated brain-specific *Atp2a2* knockout (cKO) mice by crossing *Nestin-Cre* (*NesCre*) transgenic mice with *Atp2a2^flox/flox^* mice (Fig. 1A). However, the *Atp2a2* cKO mice (*Atp2a2 ^flox/flox^*; *NesCre^tg/•^*) were not observed among the newborn pups obtained by mating between the *Atp2a2 ^flox/+^*; *NesCre^tg/•^* mice and *Atp2a2 ^flox/flox^* mice. The fact that no cKO mice were obtained in the 26 offspring from 6 litters suggests embryonic lethality of the cKO mice. When fetal pups were delivered by Caesarean section at embryonic day 14 (E14) and genotyped, cKO mice were identified at the expected ratio. However, these cKO mice were embryonically lethal with severe hemorrhages present in the brain (Arrow, Fig. 1B), though they appeared to have survived longer periods in the uterus than the heart-specific ATP2A2 KO mice (around E11) (21). Western blotting showed greatly reduced expression of ATP2A2 protein in the brain but not in the heart (Fig. 1C). In the brain-specific *Atp2a2* heterozygous knockout mice (hetero cKO, *Atp2a2 ^flox/+^*; *NesCre^tg/•^*), ATP2A2 protein expression levels in the brain were approximately half that of control mice (*Atp2a2^+/+^*; *NesCre^tg/•^*), and comparable to those of the conventional heterozygotes (*Atp2a2^+/−^*) (Fig. 1D and E). The hetero cKO appeared healthy even at 2 years after birth and their body weights were similar among genotypes (Fig. 1F, control, 45.3 ± 1.1 g; hetero cKO, 47.5 ± 1.0 g). In contrast, conventional heterozygotes showed serious somatic phenotypes and greatly reduced body weight (Fig. 1G, +/+, 51.2 ± 2.2 g; +/−, 37.1 ± 1.6 g; ^*^^*^, *P* < 0.01, two-tailed unpaired *t*-test), as observed in a previous study (13). These results showed that the brain-specific heterozygous knockout of *Atp2a2* avoided the somatic abnormalities seen in the conventional heterozygotes, allowing behavioral analyses of the hetero cKO mice to be performed. No apparent structural abnormalities were detected in the brains of the hetero cKO mice (Fig. 1H).

**Figure 2:**
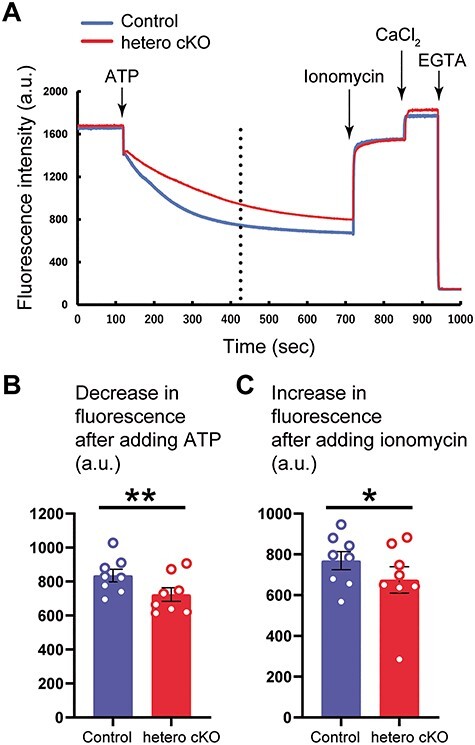
Impaired ER-Ca^2+^ uptake activity in the ER membranes isolated from the brain tissues of *Atp2a2* hetero cKO mice. (**A**) Representative time courses of the fluorescence intensity change in the buffer. Dashed vertical line: time point approximately 300 s after adding ATP. (**B**) Decrease in the fluorescence intensity after adding ATP to induce Ca^2+^ uptake into the ER (values on the dashed line in panel A were compared.) (**C**) Increase in the fluorescence intensity after adding ionomycin to estimate intra-microsomal Ca^2+^ contents. *n* = 8 samples per genotype. ^*^, *P* < 0.05; ^*^^*^, *P* < 0.01.

**Figure 3:**
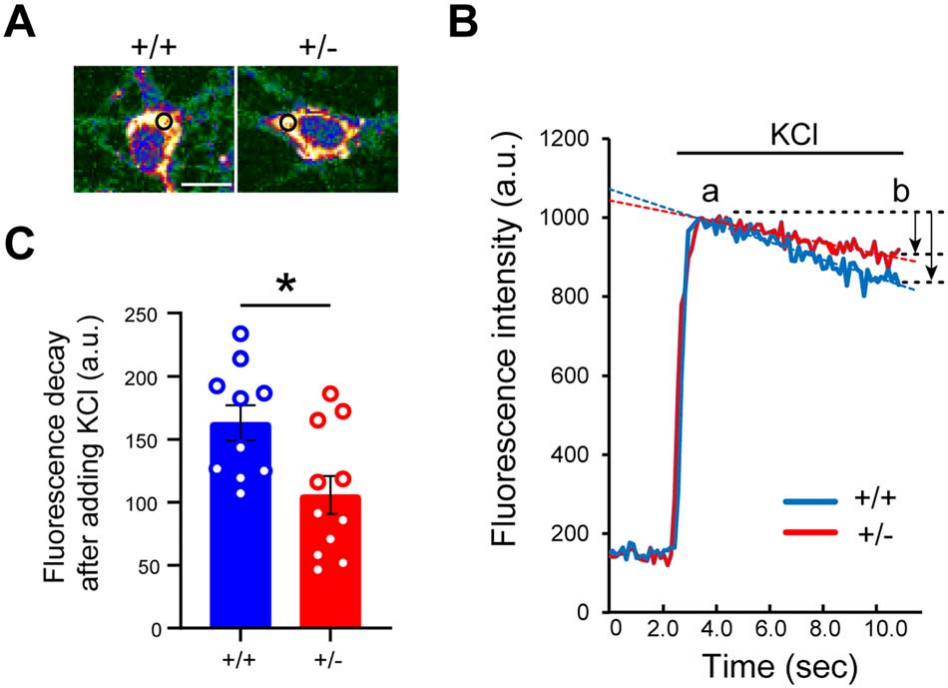
Altered Ca^2+^ dynamics in *Atp2a2* heterozygous neurons with G-CaMP7 imaging. (**A**) Representative images of the primary hippocampal neurons derived from *Atp2a2* wild-type (+/+) or heterozygous (+/−) mice expressing G-CaMP7. Pseudocolor images were created from fluorescence images. Black circles, the region of interest for fluorescence intensity analysis in a cytoplasmic region. Scale bar, 10 μm. (**B**) Representative traces of the fluorescence intensity in the cytoplasmic regions. After adding 100 mm KCl, fluorescence intensity sharply increased and gradually decreased in both genotypes. Arrows, fluorescence intensity decays between two time points (a and b, approximately 3.0 and 11.0 s, respectively). (**C**) Statistics of the fluorescence intensity decay after depolarization. Fluorescence intensity decay was difference of values between the two time points indicated in panel B (*n* = 10, +/+; *n* = 11, +/− cells). ^*^, *P* < 0.05.

To evaluate the significance of the reduced expression levels of ATP2A2 protein at the cellular level, we extracted an ER membrane fraction from the brain tissue and examined the Ca^2+^ uptake activity into the ER through ATP2A2 by spectrophotometric assay (Fig. 2A). As shown in the fluorescence intensity profiles, Ca^2+^ was rapidly imported into the ER in both genotypes after Ca^2+^ loading by adding ATP. However, the ER membranes from hetero cKO mice showed slower Ca^2+^ uptake activity (Fig. 2A). The uptake activity was most prominent around the first 300 s following the addition of ATP in both genotypes, similar to other reports (22,23). The ER membranes from hetero cKO showed significantly reduced Ca^2+^ uptake activity in the period (Fig. 2B; Control, 835.1 ± 37.0; hetero cKO, 723.9 ± 40.0 a.u.; *P* = 0.0076, two-tailed paired *t*-test). The intramembrane Ca^2+^ contents of hetero cKO samples were also reduced (Fig. 2C; Control, 769.0 ± 44.6; hetero cKO, 674.8 ± 64.9 a.u.; *P* = 0.037, two-tailed paired *t*-test). Next, we analyzed the effects of the impaired Ca^2+^ uptake activity on cytoplasmic Ca^2+^ dynamics in response to neuronal excitability (Fig. 3). We prepared primary hippocampal neurons from *Atp2a2* wild-type (+/+) and heterozygous (+/−) embryos expressing G-CaMP7 and monitored Ca^2+^ dynamics in the cytosol by measuring the fluorescence signals (Fig. 3A). We observed that the decay of the cytosolic Ca^2+^ levels after depolarization with KCl was slower in *Atp2a2* heterozygous (+/−) neurons than in the wild-type (+/+) neurons (Fig. 3B and C; +/+, 163.2 ± 14.0; +/−, 105.8 ± 15.1 a.u.; *P* = 0.0122, two-tailed unpaired *t*-test). These results suggested that heterozygous deletion of ATP2A2 caused impairment of neuronal Ca^2+^ signaling *in vivo* as well as Ca^2+^ uptake activity in the isolated ER.

**Figure 4:**
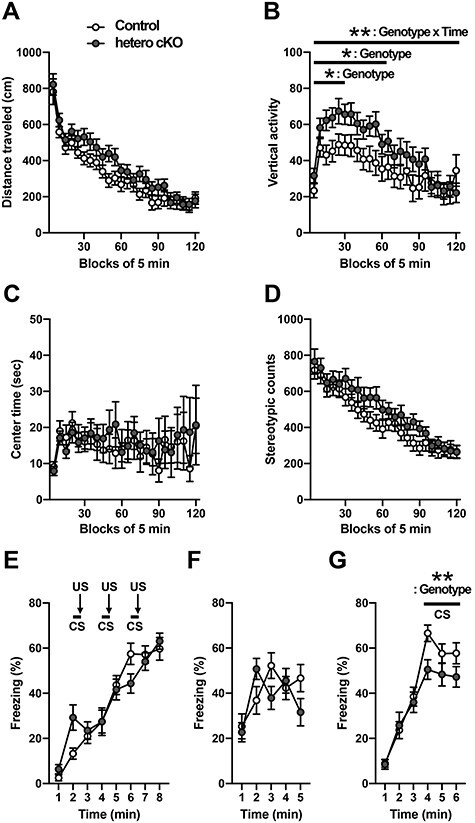
*Atp2a2* hetero cKO mice showed abnormal behaviors in novel environments and impairments in fear memory. (**A**–**D**) Open field test (*n* = 22 mice per genotype). Total distance traveled (A), vertical activity counts (B), time spent in center area (C), stereotypic counts (D). ^*^^*^, *P* < 0.01, genotype × time interaction effect; ^*^, *P* < 0.05, genotype effect (see text in detail). (**E**–**G**) Fear conditioning test (*n* = 21, control; *n* = 20, hetero cKO). Percentage of freezing during the conditioning on day 1 (E), the context test (F) and the cued test on day 2 (G). ^*^^*^, *P* < 0.01, genotype effect.

### Behavioral abnormalities in *Atp2a2* brain-specific heterozygous KO mice

To comprehensively analyze behavioral characteristics in hetero cKO mice, we performed a behavioral test battery (Fig. 4, and Supplementary Material, Figs S1–S3). For the distance traveled in an open field test for 120 min, there was no statistically significant change for the overall period, however, hetero cKO mice showed a tendency to increase for the first 60 min (Fig. 4A; first 60 min, genotype, *P* = 0.0829, *F*(1, 42) = 3.155; genotype × time, *P* = 0.718, *F*(11, 462) = 0.7222; two-way repeated measures ANOVA). The hetero cKO mice showed increased vertical activity in the open field test with a significant genotype-time interaction in a total 120 min period and a significant genotype effect for the first 60 and 30 min periods (Fig. 4B; total 120 min, genotype × time, *P* = 0.0003, *F*(23, 966) = 2.376; genotype, *P* = 0.1188, *F*(1, 42) = 2.536; first 60 min, genotype × time, *P* = 0.7567, *F*(11, 462) = 0.6813; genotype, *P* = 0.0266, *F*(1, 42) = 5.279; first 30 min, genotype × time, *P* = 0.2558, *F*(5, 210) = 1.322; genotype, *P* = 0.0278, *F*(1, 42) = 5.198; two-way repeated measures ANOVA). No significant changes were found in the center time and stereotypic counts (Fig. 4C and D). To assess the ability to learn and remember an association between environmental cues and aversive experiences, a contextual and cued fear conditioning test was performed (Fig. 4E–G). The hetero cKO mice showed similar levels of freezing behavior compared to control mice in a contextual fear test (Fig. 4F) but they showed impaired fear memory in a cued fear test (Fig. 4G, last 3 min; genotype, *P* = 0.0079, genotype × time, *P* = 0.661; two-way repeated measures ANOVA). In other tests that examined sensorimotor functions, learning and memory performance, anxiety-like behaviors, depression-like behaviors and social interaction, no significant differences were observed (Supplementary Material, Figs S1–S3). Electroencephalographic (EEG) recordings were also carried out for 3 days, but no marked epileptic waveforms were observed in the EEG of hetero cKO mice and the sleep/wake cycles deduced from the analysis of the EEG frequency were not significantly different between genotypes (Supplementary Material, Fig. S4).

**Figure 5:**
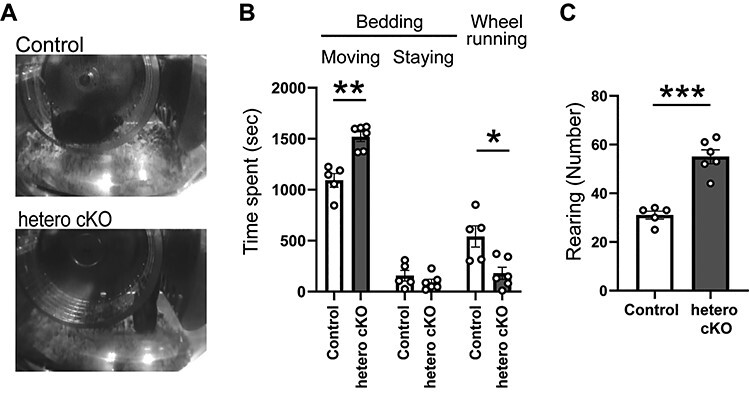
*Atp2a2* hetero cKO mice showed altered behavioral response after cage changing in the wheel running activity. (**A**–**C**) Video analysis of the mouse behaviors for 30 min after cage changing. Video images of the mice (A). Classification of the mouse behaviors after cage changing (30 min; 1800 s); the mouse was on the wheel (Wheel running), moving (Moving) or staying on the bedding (Staying) (B). Vertical activity (rearing) counts for 30 min after cage changing (C). ^*^, *P* < 0.05; ^*^^*^, *P* < 0.01; ^*^^*^^*^, *P* < 0.0001; two-tailed unpaired *t*-test.

We measured wheel running activity for more than 3 months to evaluate whether the hetero cKO mice would show behavioral changes relevant to bipolar disorder or recurrent depression (Supplementary Material, Fig. S5) (24,25). The duration or pattern of the wheel running activities appeared similar among genotypes (*n* = 15 mice for each genotype: Supplementary Material, Fig. S5A). Generally, mice show an increase in wheel running activity immediately after cage changing (every 2 weeks), which was similarly observed in the control mice. However, this behavioral response was not observed in the hetero cKO mice (Supplementary Material, Fig. S5A–C). On the other hand, behaviors other than wheel running activity were not clarified with the equipment. To analyze the behavioral difference after the cage changing more closely, video recordings were performed with a pair of control and hetero cKO mice for 90 min following cage changing over 3 months (Fig. 5; *n* = 5 and 6 cage changings for control and hetero cKO, respectively). After the cage changing, for the first 30 min (corresponding to 1800 s), the hetero cKO mouse spent more time moving on the bedding (Fig. 5B; control, 1092.2 ± 151.4 s; hetero cKO, 1519.8 ± 117.3 s; *P* = 0.0005, two-tailed unpaired *t*-test) and accordingly it showed a reduced amount of wheel running activity compared to the control mouse. Both genotypes spent similar time in staying behavior on the bedding (Fig. 5B), in which the mice appeared apparently awake but did not move vigorously and kept staying on the bedding. The hetero cKO mouse showed significantly increased vertical activity (or rearing) after the cage changing (Fig. 5C; control, 31 ± 1.6; hetero cKO, 55 ± 2.8; *P* < 0.0001, two-tailed unpaired *t*-test), which was consistent with the result in the open field test (Fig. 4B). Collectively, these results suggest that the hetero cKO mice had altered behavioral responses to novel environments.

### Extracellular DA levels were elevated in the NAc of *Atp2a2* brain-specific heterozygous KO mice

**Figure 6:**
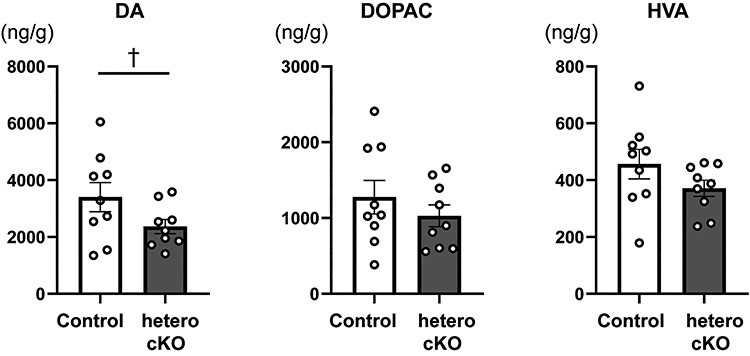
Tissue contents of DA and its metabolites in the NAc of *Atp2a2* hetero cKO mice. Tissue contents of DA, DOPAC, and HVA in the NAc following open field tests for 30 min. *n* = 9 mice per genotype. C, control. ^†^, *P* = 0.089.

The increase of vertical activity in novel environments and impaired fear memory (Figs 4 and 5) could be correlated with the dysregulation of the DA system in the NAc (26,27). We performed monoamine analysis using tissue homogenates of brain samples. As shown in the open field tests in Figure 4B, rearing scores in hetero cKO mice were markedly increased during the first 30 min. Thus, hetero cKO mice were subjected to open field tests for 30 min, then sacrificed, and NAc tissues were collected and homogenized. DA and its metabolites (DOPAC and HVA) were quantified by HPLC coupled with electrochemical detection (Fig. 6). DA levels tended to be decreased in the NAc of the hetero cKO mice (Fig. 6, left panel; control, 3401.6 ± 513.1; hetero cKO, 2368.8 ± 249.3 ng/g, *P* = 0.089, two-tailed unpaired *t*-test). However, there were no significant changes in the DOPAC and HVA levels.

**Figure 7:**
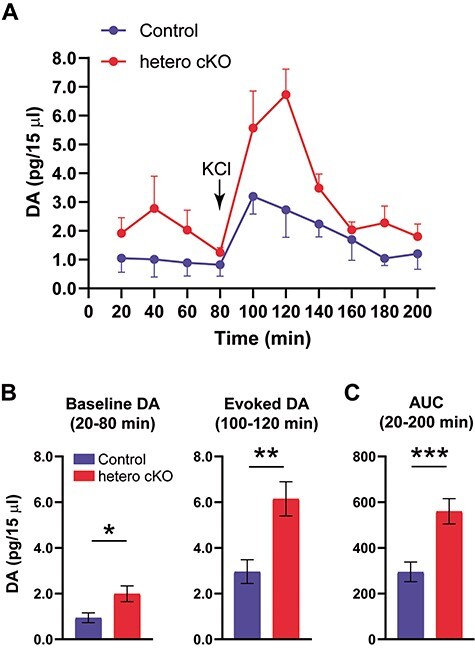
Extracellular DA levels were increased in the NAc in *Atp2a2* hetero cKO mice. (**A**) Time course of the extracellular dopamine levels with microdialysis. (**B**) Left, baseline dopamine levels (average of 20–80 min). Right, evoked dopamine levels after high K^+^ stimulation (average of 100–120 min). (**C**) Area under the curve (AUC) values from 20–120 min. *n* = 3 mice per genotype. ^*^, *P* < 0.05; ^*^^*^, *P* < 0.01; ^*^^*^^*^, *P* < 0.001.

To evaluate DA levels in the NAc *in vivo*, we examined the extracellular DA levels with microdialysis (Fig. 7A). Baseline levels of DA were significantly increased (Fig. 7B, left; control, 0.94 ± 0.21; hetero cKO, 1.99 ± 0.35 pg/15 μl; *P* = 0.017, two-tailed unpaired *t*-test). Moreover, the increase became more evident after high K^+^ stimulation (Fig. 7B, right; control, 2.96 ± 0.74; hetero cKO, 6.15 ± 0.52 pg/15 μl; *P* = 0.0056, two-tailed unpaired *t*-test). Area under the curve (AUC) analysis also showed that extracellular DA levels were significantly increased in hetero cKO mice during the overall period (Fig. 7C; 295.2 ± 43.06, control, 560.3 ± 55.24, hetero cKO; *P* = 0.0005, two-tailed unpaired *t*-test). These results suggested a hyperdopaminergic state in the NAc of hetero cKO mice.

## Discussion

We generated and analyzed *Atp2a2* brain-specific heterozygous KO mice to model bipolar disorder and schizophrenia. This is the first report analyzing *in vivo* significance of ATP2A2 in the central nervous system through a conditional gene-targeting approach. We found that *Atp2a2* heterozygous neurons showed a slower decay of the cytosolic Ca^2+^ levels, which confirmed an essential role of the ATP2A2 pump for Ca^2+^ homeostasis in neurons. Defective DA neurotransmission was observed in the NAc of hetero cKO mice, which suggested a hyperdopaminergic state. *Atp2a2* brain-specific hetero cKO mice showed altered behavioral responses to novel environments and impairments in fear memory, which were consistent with mental disorders. However, other phenotypic features related to mood disorders and schizophrenia were absent, which included deficits in prepulse inhibition, working memory, and social behaviors. The genetic background of mutant mice and flanking genes around the targeted locus have been shown to have striking effects, especially when performing behavioral analyses of mouse models of mood disorders and schizophrenia (28,29). Moreover, gene expression, anatomical and electrophysiological phenotypes are also drastically altered by genetic background (30). Previously reported *Atp2a2* heterozygous mice were described to have a mixed genetic background of 50% 129/SvJ and 50% Black Swiss (13). However, heterozygous mice are expected to carry more alleles of 129/SvJ and wild-type littermates carry more alleles of Black Swiss near the deleted gene, i.e. flanking gene problem. In other words, the heterozygous and wild-type mice are systematically different in genetic backgrounds, thereby making it difficult to perform and interpret behavioral analyses with these mice. The fact that Swiss Black is an outbred strain may further complicate situations. In the present study, the *Atp2a2* mutant mice were originally established using C57BL/6N derived embryonic stem cells with homologous recombination (31) and have been maintained in a C57BL/6N background in our facility. Therefore, their genetic background is relatively clear and phenotypic variations dependent on flanking genes would be minimal in the behavioral abnormalities we observed in the hetero cKO mice. However, the possibility that this homogeneous genetic background of C57BL/6N modified or suppressed phenotypic expression still exists (30). Some phenotypes (e.g. prepulse inhibition, working memory, and social behaviors) might be more susceptible to such suppressant effects than others (e.g. fear memory) in the hetero cKO mice. Another possibility that needs to be considered is whether heterozygosity is equally detrimental in mice and humans. The mouse might be more tolerant to gene dosage reductions than humans. However, in this study, homozygous deletion of ATP2A2 in the whole brain by *NesCre* transgene resulted in embryonic lethality (Fig. 1B).

Increasing DA transmission by using a dopamine transporter (DAT) inhibitor showed an increase in vertical activity (26), while blockade of DA neurotransmission by a dopamine D1 receptor antagonist showed a reduction in vertical activity in rodents (32). However, these models showed changes in vertical activity that accompanied overall changes in locomotor activity. In contrast, locomotor activity was not significantly changed in the *Atp2a2* hetero cKO mice, though the effect was not acute but chronic and would be milder than that in the studies using drugs. Some genetically engineered mouse models showing spontaneous hyperdopaminergic states could be useful to interpret the results in the hetero cKO mice. DAT KO mice had a 5-fold elevation in the extracellular basal levels of DA compared to wild-type and showed increased locomotor activity, while DAT heterozygotes had a 2-fold elevation in the extracellular basal levels of DA but showed no significant changes in locomotor activity (33,34). However, DAT heterozygotes showed some characteristic behavioral abnormalities including associative memory (35,36). DAT A559V heterozygous knock-in mice had a 7- to 8-fold elevation in the extracellular basal levels of DA, while they showed no significant change in locomotor activity but showed significantly reduced vertical activity (37). The point mutation was derived from patients with mental disorders including bipolar disorder. Considering these conflicting results, the paradoxical findings in the hetero cKO mice, i.e. a 2-fold elevation in the extracellular DA levels (Fig. 7) and no significant change in locomotor activity associated with the abnormal vertical activity (Fig. 4), are also compatible with the findings from other studies. Impairments in fear memory have also been suggested to be related to increased levels of DA in the NAc (27) and thus are relevant to the similar finding observed in human subjects with schizophrenia (38). Thus, increased levels of extracellular DA in the NAc (Fig. 7) may explain a causal relationship between *ATP2A2* and mental illness.

Though extracellular DA levels measured by microdialysis were increased, tissue DA levels were not significantly different in this study. Some reports have also shown similar significant differences in extracellular DA levels in the microdialysis without apparent changes in DA levels in the tissue homogenates (37,39). These discrepancies would be partly due to differences in the measurement principles. Microdialysis primarily measures DA concentrations in the extracellular space, whereas in the tissue homogenates DA molecules present in intracellular compartments are also quantified alongside residual extracellular DA. Extracellular DA levels measured by microdialysis would be more appropriate to interpret behavioral phenotypes rather than levels in the tissue homogenates.

DA release was elevated in *Atp2a2* hetero cKO mice, as might be expected of mice in which Ca^2+^ transients were prolonged. In the presynaptic terminal, the vesicular release was shown to be efficiently regulated via ER-Ca^2+^ uptake activity (40). A significant increase in mIPSC frequency, not amplitude, via the presynaptic release of GABA in mouse cerebellar Purkinje cells, was shown to be caused by blocking the activity of the presynaptic ATP2A2 pump with cyclopiazonic acid or thapsigargin (41). It will be important to address how the reduced expression of ATP2A2 affected specifically the DA system in the hetero cKO mice. It may be related to an intrinsic mechanism of DA release in the ventral tegmental area (VTA), as DA neurons have two different release modes, phasic and tonic (42–45). These firing patterns of DA neurons may make them selectively sensitive, compared to other neuronal cell types, in deficiencies of ER-Ca^2+^ uptake activity in the hetero cKO mice. It may also need to be considered that DA release in the NAc can be dynamically modulated without corresponding changes in the spiking of the VTA DA neurons (46). In addition, compensation by increased expression of plasma membrane Ca^2+^ ATPase pumps was suggested to have a role in maintaining homeostasis in the vesicular release in *Atp2a2* heterozygous pancreatic acini (47). Such a compensatory mechanism could be insufficient in the DA nerve terminals of the hetero cKO mice.

Some patients of Darier’s disease have been reported to have epilepsy in addition to psychotic symptoms (48–50). To examine this, we performed EEG recording but no remarkable epileptic patterns were observed in the waveforms in the hetero cKO mice and the sleep/wake cycles were not significantly different among genotypes (Supplementary Material, Fig. S4). Abnormalities in EEG delta waves during sleep have been reported in schizophrenia patients (51) and its relevance with *ATP2A2* has been implicated (52). Thus, a more detailed analysis of EEG could help characterize the neuronal network activity of the *Atp2a2* hetero cKO mice.

Among the Darier’s disease patients with LOF mutations in *ATP2A2*, approximately 20% of patients showed psychoses (11). This implies that interactions between LOF mutations in *ATP2A2* and other genetic factors, as well as environmental/epigenetic factors, could promote the onset of the disorders. Though many useful mice models for mood disorders and schizophrenia have already been established (1,25,53–55), most models exhibit only a few aspects of the disorders, suggesting involvement of multiple genetic factors. It might be beneficial to generate compound mutant mice of *Atp2a2* hetero cKO with other established model mouse lines, which could make the phenotypic features more evident. Collectively, our results show that the *Atp2a2* hetero cKO mice could be a useful model for some facets of mood disorders and schizophrenia along with other currently used mouse models.

## Materials and Methods

### Generation of the *Atp2a2* conditional knockout mice

*Atp2a2* mutant mice (*Atp2a2^tm1a(EUCOMM)Hmgu^*) were obtained from the European Conditional Mouse Mutagenesis (EUCOMM) program, which were originally generated by homologous recombination with C57BL/6N-derived embryonic stem cells (31). The allele contained *lacZ* and *neo* cassettes inserted in the intron, which was expected to disrupt gene expression and result in a knockout allele, and hence the mouse was regarded as an *Atp2a2* heterozygous mouse (KO first allele; hereafter referred to *Atp2a2^+/−^*). The heterozygous mice have been maintained by crossing with C57BL/6N mice (CLEA Japan Inc.) in our facility, thus they were coisogenic mice with C57BL/6N background. These mice were crossed with *FLPe* transgenic mice, which had C57BL/6J background (56), to generate mice in which *Atp2a2* was flanked by loxP sites. The *Atp2a2 ^flox/+^* mice were crossed with the *NesCre* transgenic mice (B6.Cg-Tg(Nes-cre)1Kln/J [The Jackson Laboratory]; backcrossed and maintained as C57BL/6J congenic strain in our facility), and then backcrossed with C57BL/6N mice three generations (used for behavioral test battery and wheel running activity measurement; Figs 4 and 5) and eight or more generations (used for monoamine and *in vivo* dialysis; Figs 6 and 7). Considering the crossing with the *Flpe* transgenic mouse (C57BL/6J background) to obtain the *Atp2a2 ^flox/+^* mice, the three and eight generations of backcrossing with C57BL/6N would be expected to reduce genomic material from the two deleter strains, the *Flpe* and the *NesCre* transgenic mice, to approximately less than 10% and 0.3%, respectively, while avoiding the flanking gene problem.

All animal experiment protocols were approved by the RIKEN Wako Animal Experiment Committee and all experiments were performed in accordance with the approved guidelines and regulations. All other experimental procedures were approved by the RIKEN Wako Safety Center and were carried out in accordance with the approved guidelines.

### Western blotting

Tissues (brain and heart) were homogenized with a Potter-type homogenizer in ice-cold buffer (25 mm Tris–HCl, 150 mm NaCl, 1% NP-40, 0.1% SDS) containing 1× cOmplete Mini protease inhibitor cocktail (Roche). After centrifugation, supernatants were recovered, and protein concentrations were determined using a BCA kit (Thermo Fisher). Proteins were separated by SDS-PAGE, transferred to PVDF membranes, and subjected to western blotting with antibodies for ATP2A2 (sc-8094 or 8095, Santa Cruz Biotechnology) and β-actin (A5441, Sigma-Aldrich).

### Histological analysis

Male mice (12 months old) were transcardially perfused with PBS and fixed with paraformaldehyde (PFA). The fixed samples were dehydrated with ethanol, followed by xylene, and embedded in paraffin. Blocks were cut using a microtome (SM2000R, Leica) into 10 μm serial sections, and mounted onto glass slides, deparaffinized, and subjected to Nissl staining.

### ER-Ca^2+^ uptake activity assay

The assay was performed as previously described (22). Briefly, to prepare microsomes containing ER membranes, cerebellum tissues were dissected from male mice aged 3–4 months. They were homogenized in a buffer (0.25 M sucrose, 1 mm EGTA, and 50 mm HEPES at pH 7.4) supplemented with protease inhibitor cocktail Halt (Thermo Fisher), centrifuged for 15 min at 4000*g* at 4°C, and the supernatants were centrifuged for 30 min at 100 000*g* at 2°C. The supernatants were homogenized again and centrifuged for 30 min at 100 000*g* at 2°C, and the pellets (microsome fractions) were resuspended in the buffer and kept at −80°C until the assay. Ca^2+^ uptake activity was monitored by measuring the fluorescence of Calcium Green-1 using a spectrophotometer F-2500 (Hitachi). Microsomes were diluted to 600 μl with mobilization buffer containing (in mm: KCl, 110; NaCl, 10; KH_2_PO_4_, 5; MgCl_2_, 2; HEPES, 50 at pH 7.2) supplemented with 2 μm calcium green-1, 10 mm phosphocreatine, 40 U/ml creatine kinase, 1 mm DTT, and 2.5 μg/ml oligomycin. Ca^2+^ uptake to microsomes was initiated by adding 0.1 M ATP. After 600 s, 10 μm ionomycin was added to estimate the intra-microsomal Ca^2+^ content. Before ending the experiment, 100 μm CaCl_2_ and 10 mm EGTA were added to get *F*_max_ and *F*_min_, respectively.

### Ca^2+^ imaging in primary hippocampal neurons with G-CaMP7

To obtain *Atp2a2* wild-type and heterozygous neurons expressing G-CaMP7 (*Atp2a2^+/+^; G-CaMP7^tg/•^* and *Atp2a2^+/−^; G-CaMP7^tg/•^*, respectively), *Atp2a2* heterozygotes were crossed with *G-CaMP7* transgenic mice (G7NG817 line) (57). G-CaMP7 expressing hippocampal neurons were prepared from E16.5 mouse embryos according to a standard procedure (58) and cultured in chambered cover glasses. The cells were placed in a thermostatic chamber (Tokai Hit) attached to an inverted laser scanning confocal microscope (FV-3000RS, Olympus) equipped with a resonant scanner and an oil-immersion objective lens (UPLSAPO 40XS). The cells were stimulated with 100 mm KCl to become depolarized. The images were acquired at a frequency of 100 images/s and analyzed. The region of interest (ROI) was manually selected by placing a circular ROI in a cytoplasmic region in the cell body excluding the nucleus. Procedures for imaging with depolarization were established based on the previous reports (59,60), in which ER-Ca^2+^ uptake in neurons efficiently worked within seconds after depolarization. Fluorescence intensity decay was defined as the difference of the fluorescence intensities between two time points after depolarization (see legend of Fig. 3 for detail).

### Behavioral test battery

The behavioral test battery was performed with male mice at the following ages; 3–5 months old for open field test, rotarod test, hot plate test, startle response/prepulse inhibition test, light/dark transition test, elevated plus maze test, Porsolt forced swim test, social interaction test in a novel environment, sociability and social novelty preference test; 6–11 months old for fear conditioning test, Barnes maze test, T-maze spontaneous alternation test, Tail suspension test, and social interaction test in a home cage. Methods and results for analyses other than the open field and fear conditioning tests are described in Supplementary Material section.

### Open field test

An open field apparatus (40 × 40 × 30 cm, VersaMax system, AccuScan Instruments) was used, which was illuminated at 100 lx. Total distance traveled, vertical activity (rearing, measured by counting the number of photobeam interruptions), time spent in the center area, and stereotypic counts were recorded for 120 min.

### Cued and contextual fear conditioning test

A cued and contextual fear conditioning test was performed as previously described (61). A conditioned stimulus (CS, 55 dB white noise) was presented for 30 s and paired with the unconditioned stimulus (US, 0.3 mA foot shock in the last 2 s of CS). Each mouse received the three CS-US pairings with 2 min intervals on day 1. Twenty-four hours later (On day 2), contextual testing was carried out in the same test chamber for 5 min without CS and US. Subsequent cued testing was carried out 3 h later with altered context (in a novel triangular test chamber) for 6 min. Freezing was recorded during the conditioning, contextual testing and cued testing sessions.

### Wheel running activity measurement

Long-term recording of wheel running activity was performed as described previously (24,25). Briefly, male mice (3 months old) were individually housed in cages equipped with a running wheel. The day when the mice were transferred to the cages from conventional breeding cages was designated as day 1, and thereafter cage changings were performed every 2 weeks. Light/dark (12:12 h) cycles were controlled by a PC system, and wheel running activity was recorded by an on-line computer system (O’HARA & CO.). Food and water were constantly available *ad libitum*. Actograms were drawn using an ImageJ (Fiji) plug-in ‘ActogramJ’ (62). Video recordings were carried out with a DVD MovieWriter 7 (Corel Corporation).

### Monoamine analysis

Male mice (3–4 months old) were individually placed in an open field apparatus (60 × 60 × 30 cm; O’HARA & CO.), which was illuminated at 70 lx. Following 30 min in the apparatus, mice were removed and euthanized by cervical translocation. Whole brains were quickly removed and NAc tissues were excised into ice-cold saline, weighed, frozen with liquid nitrogen, and stored at −80°C. Tissues were deproteinized, homogenized in a perchloric acid solution, and centrifuged followed by neutralization and HPLC analysis coupled with electrochemical detection as previously described (55,63).

### Microdialysis

Male mice (12–17 months old), from the same cohort used in the monoamine analysis, were anesthetized with isoflurane and placed into a stereotaxic frame. A guide cannula (CXG-6, EiCOM) was implanted into the NAc (coordinates relative to bregma in mm, AP +1.3, ML +0.8, and DV −3.9) and fixed onto the skull using acrylic resin PROVINICE (SHOFU INC.). Mice were left to recover for at least 1 week. On the sampling day, a microdialysis probe with an active length of 1 mm (CX-1-6-01, EiCOM) was inserted into the guide cannula. The mouse was placed in a chamber (clear cylindrical container, 14 cm diameter, 22 cm high, Instech Laboratories, Inc.) and continuously perfused with artificial cerebrospinal fluid (aCSF, containing in mm: NaCl, 148; KCl, 4; CaCl_2_, 1.2; MgCl_2_, 0.85) via a syringe pump (CMA/100, Harvard Apparatus) at a flow rate of 1 μl/min for at least 2 h before baseline sampling. Dialysates were collected every 20 min using a refrigerated fraction collector (820, Univentor) at 2°C with plastic microvials preloaded with perchloric acid to minimize degradation of DA. High potassium stimulation was carried out by perfusing aCSF with 60 mm KCl using a liquid switch (CMA/110, Harvard Bioscience, Inc.). Dialysate samples were kept at −80°C until use and DA concentration was determined by HPLC coupled with electrochemical detection.

Following microdialysis experiments, mice were anesthetized by isoflurane and transcardially perfused with saline followed by 4% PFA. Brains were dissected, post-fixed in PFA, and sectioned at 50 μm with a vibratome. The sections were mounted onto the slides and stained with hematoxylin and eosin to identify the position of the microdialysis probe. Only mice with probes located in the NAc were included in the analyses.

## Experimental Design and Statistical Analysis

Statistical significance was analyzed using GraphPad Prism (GraphPad Software) or Statcel (OMS Publishing Co.). The number of animals used (*n*) and the specific statistical tests used are indicated in the corresponding part of the main text or figure legends for each experiment. All data are presented as mean ± SEM.

## Supplementary Material

ATP2A2_Fig_S1_ddab137

ATP2A2_Fig_S2_ddab137

ATP2A2_Fig_S3_ddab137

ATP2A2_Fig_S4_ddab137

ATP2A2_Fig_S5_ddab137

Nakajima_Supplementary_Texts_210505_ddab137
