# Correction to: Brain-specific heterozygous loss-of-function of ATP2A2, endoplasmic reticulum Ca^2+^ pump responsible for Darier's disease, causes behavioral abnormalities and a hyper-dopaminergic state

**DOI:** 10.1093/hmg/ddad151

**Published:** 2023-09-14

This is a correction to: Kazuo Nakajima, Mizuho Ishiwata, Adam Z Weitemier, Hirotaka Shoji, Hiromu Monai, Hiroyuki Miyamoto, Kazuhiro Yamakawa, Tsuyoshi Miyakawa, Thomas J McHugh, Tadafumi Kato, Brain-specific heterozygous loss-of-function of ATP2A2, endoplasmic reticulum Ca^2+^ pump responsible for Darier's disease, causes behavioral abnormalities and a hyper-dopaminergic state, *Human Molecular Genetics*, Volume 30, Issue 18, 15 September 2021, Pages 1762–1772, https://doi.org/10.1093/hmg/ddab137

In the originally published version of this manuscript, in Figure 1A, the targeted exon of ATP2A2 flanked by loxP was labelled as exon 12, instead of exon 5.

The corrected Figure 1A is provided below:

**Figure 1A:**
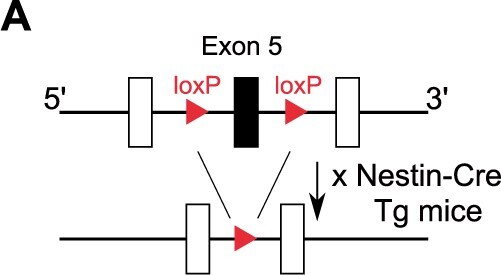


These details have been corrected only in this correction notice to preserve the published version of record.

