# Supplementary figures and images for: Brain-specific heterozygous loss-of-function of ATP2A2, endoplasmic reticulum Ca2+ pump responsible for Darier’s disease, causes behavioral abnormalities and a hyper-dopaminergic state
Source: Hum Mol Genet. 2021 Jun 8;30(18):1762–72. doi: 10.1093/hmg/ddab137 (PMC8411987; doi:10.1093/hmg/ddab137)

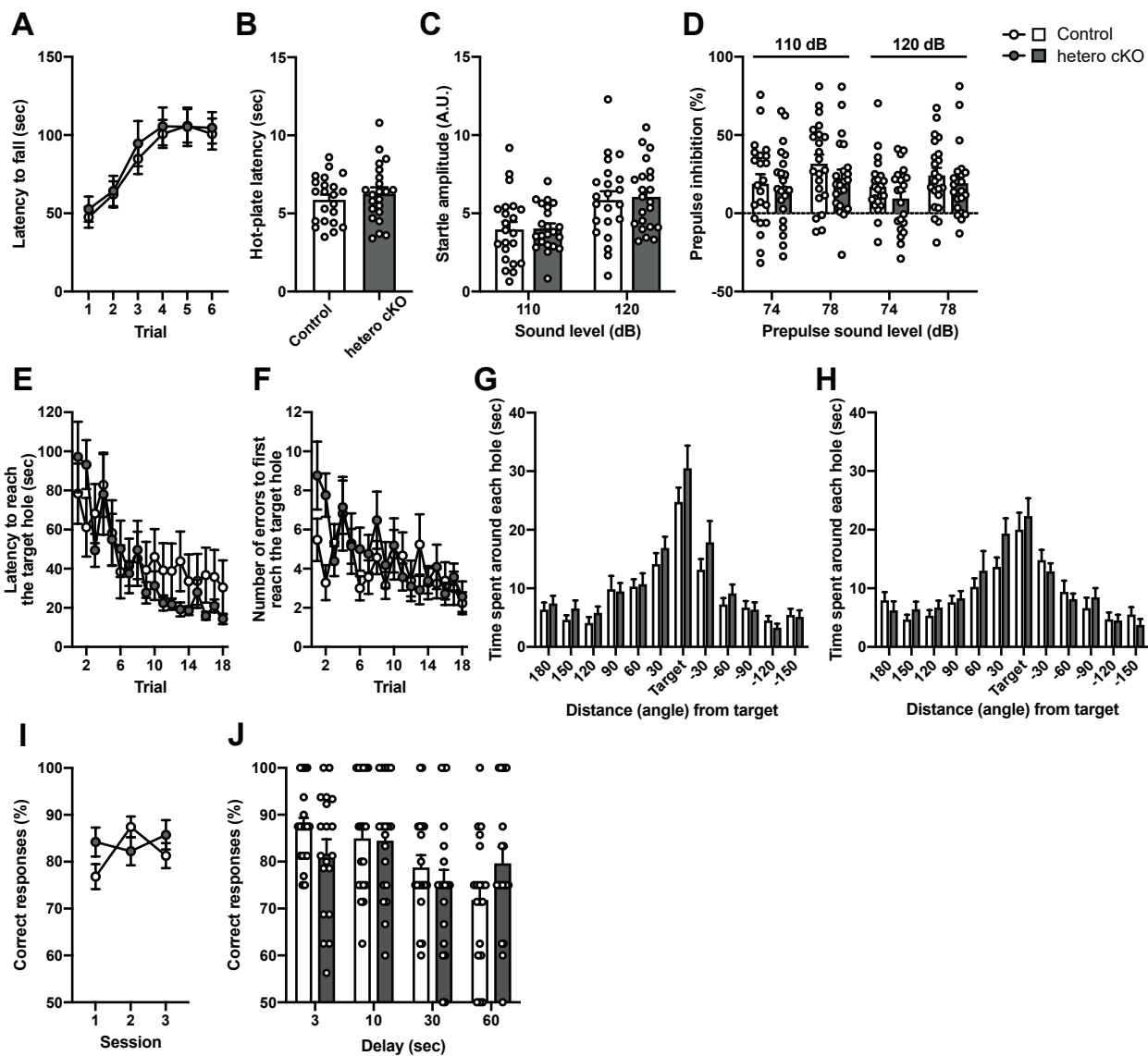

Figure S1

Supplement: ATP2A2_Fig_S1_ddab137 [file atp2a2_fig_s1_ddab137.pdf]

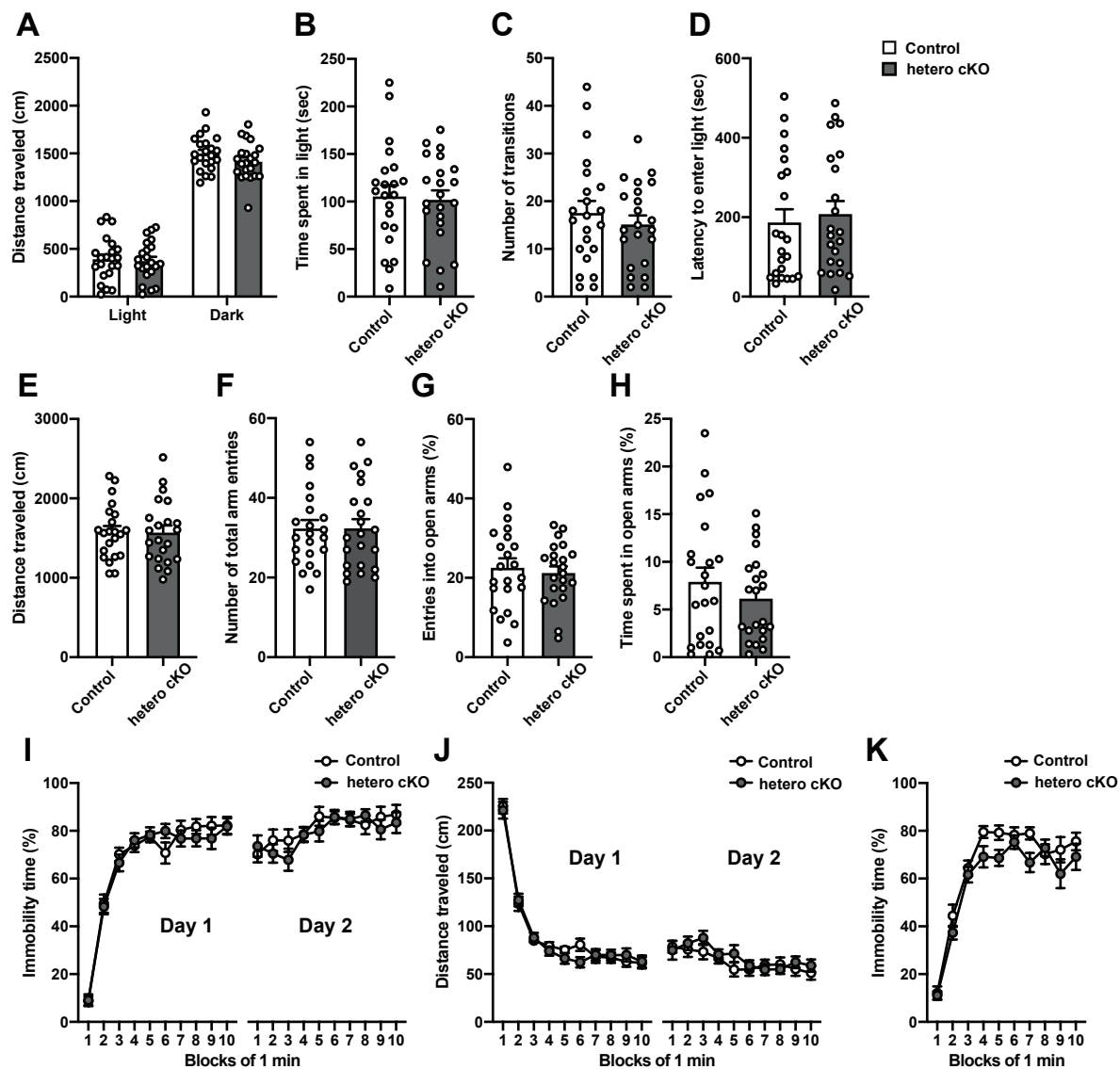

Figure S2

Supplement: ATP2A2_Fig_S2_ddab137 [file atp2a2_fig_s2_ddab137.pdf]

**A**

Control

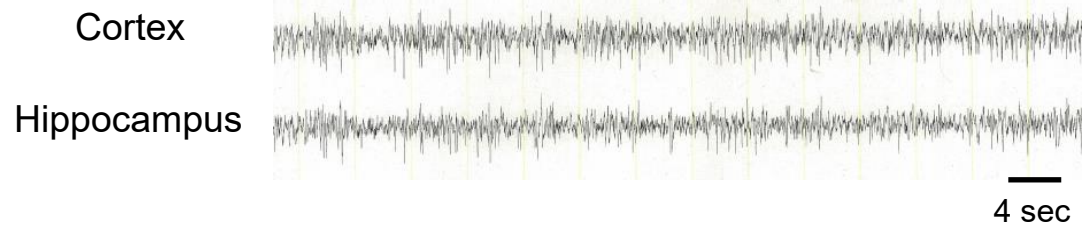

hetero cKO

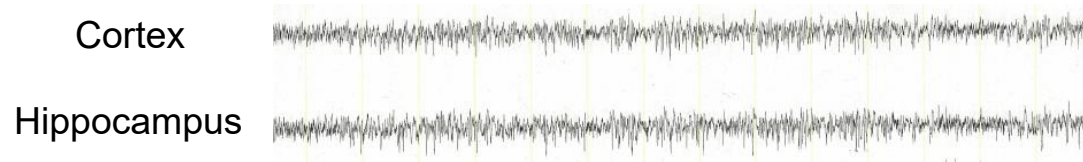

**B**

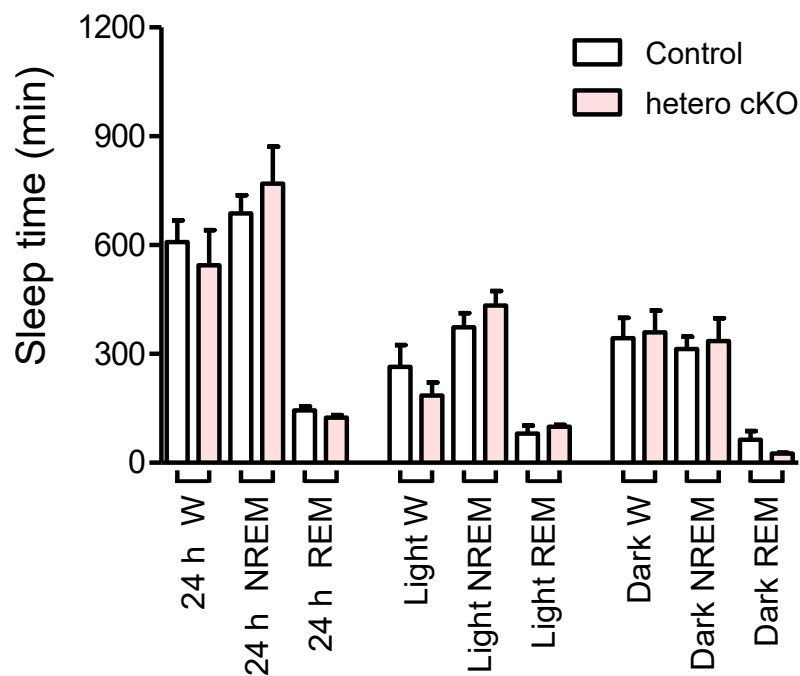

Figure S4

Supplement: ATP2A2_Fig_S4_ddab137 [file atp2a2_fig_s4_ddab137.pdf]

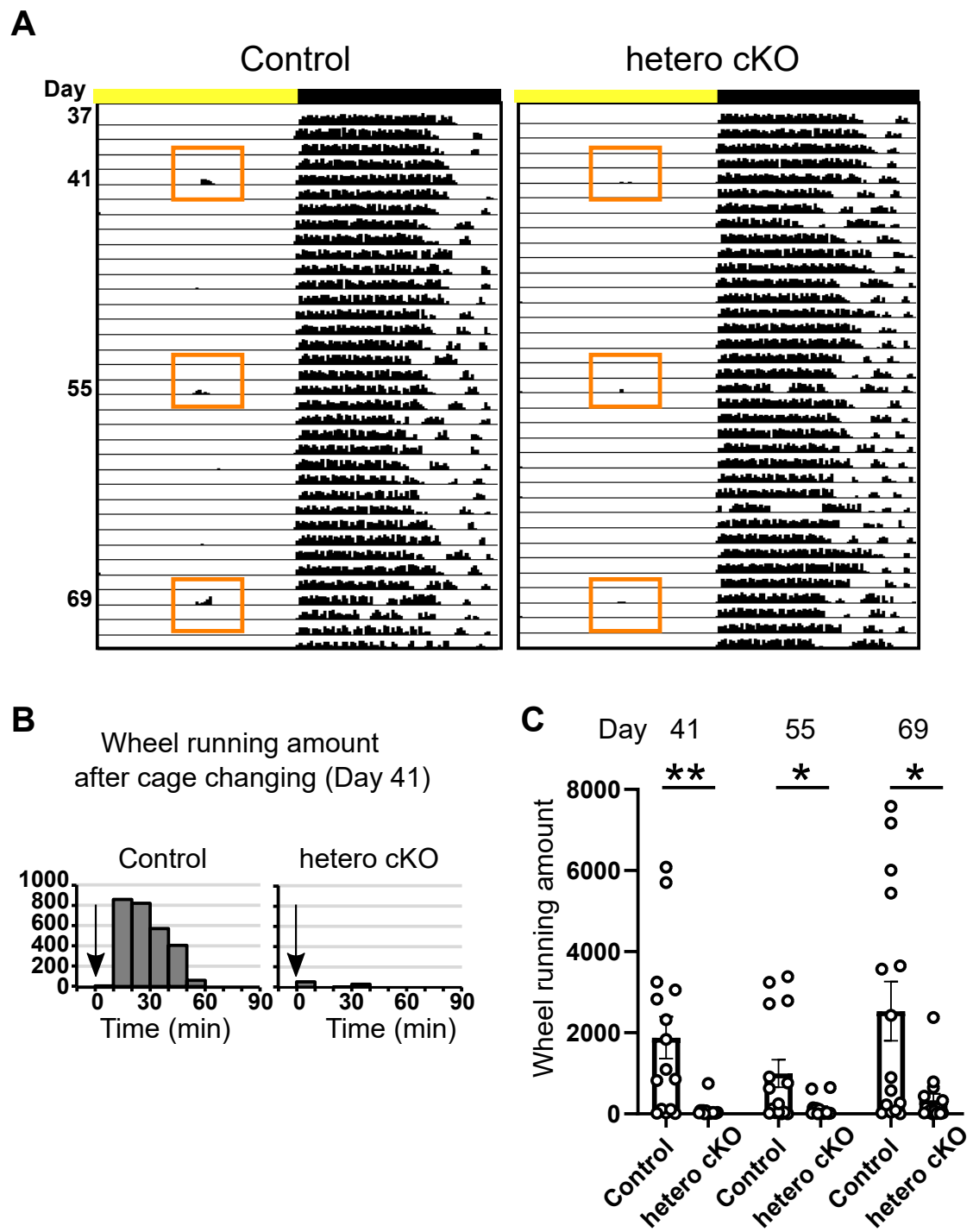

Figure S5

Supplement: ATP2A2_Fig_S5_ddab137 [file atp2a2_fig_s5_ddab137.pdf]
