# Supplementary material for: Brain-specific heterozygous loss-of-function of ATP2A2, endoplasmic reticulum Ca2+ pump responsible for Darier’s disease, causes behavioral abnormalities and a hyper-dopaminergic state
Source: Hum Mol Genet. 2021 Jun 8;30(18):1762–72. doi: 10.1093/hmg/ddab137 (PMC8411987; doi:10.1093/hmg/ddab137)
Supplement: Nakajima_Supplementary_Texts_210505_ddab137 [file nakajima_supplementary_texts_210505_ddab137.doc]

**Supplementary materials**

**Supplementary Materials and Methods**

**Rotarod test**

Motor coordination and balance were tested with the rotarod test. This test, which uses an accelerating rotarod (UGO Basile), was performed by placing mice on rotating drums (3 cm diameter) and measuring the time mice were able to maintain its balance on the rods. The speed of the rotarod accelerated from 4 to 40 rpm over a 5 min period.

**Hot plate test**

To evaluate sensitivity to a painful stimulus, the hot plate test was performed. Mice were placed on a 55.0°C (± 0.3°C) hot plate (Columbus Instruments), and latency to the first fore- or hind-paw response was recorded. The paw response was defined as either a paw lick or a foot shake.

**Startle response/Prepulse inhibition test**

To measure acoustic startle response and prepulse inhibition, a startle reflex measurement system was used (O’HARA & Co., LTD.). Mice were placed in the plastic cylinder where they were left undisturbed for 10 min to habituate just before the test. White noise (40 ms) was used as the startle stimulus. The startle response was recorded for 400 ms (measuring the response every 1 ms), starting with the onset of the startle stimulus. The background noise level in each chamber was 70 dB. The peak startle amplitude recorded during the sampling window was used as the dependent variable. A test session consisted of 6 trial types (i.e., 2 types for startle stimulus-only trials, and 4 types for prepulse inhibition trials). The intensity of the startle stimulus was 110 or 120 dB. The prepulse sound was presented 100 ms before the startle stimulus, and its intensity was 74 or 78 dB. Four combinations of prepulse and startle stimuli were used (74-110, 78-110, 74-120, and 78-120 dB). Six blocks of the 6 trial types were presented in a pseudo-random order, such that each trial type was presented once within a block. The average inter-trial interval was 15 s (range 10-20 s).

**Barnes maze test**

The Barnes maze task was conducted on “dry land”, a white circular surface that was 1.0 m in diameter, with 12 holes equally spaced around the perimeter (O’HARA & Co., LTD.). A black Plexiglass escape box (17 × 13 × 7 cm) with paper cage bedding on its bottom was located under one of the holes. The hole above the escape box represented the target, which was analogous to the hidden platform in the Morris water maze task. The location of the escape box (target) was consistent for a given mouse but was randomized across mice. The mice that could not find the box were guided to it and allowed to enter it to remain there for 30 s. One or two trials per day were conducted for 11 days. Each trial ended when the mouse entered the escape box or after 5 min had elapsed. The data were recorded by ImageBM software. On day 12 a probe test was conducted for 180 s without the escape box to assess memory based on distal environmental room cues. Another probe trial was conducted 30 days after the last training session to evaluate memory retention. The time spent around the target hole was recorded in these probe tests by the software.

**T-maze spontaneous alternation test**

The spontaneous alternation test was conducted using an automated T-maze apparatus (O’HARA & Co., LTD.). It was constructed of white plastic runways with walls 25 cm high. The maze was partitioned off into 6 areas by sliding doors that could be opened downward. The stem of the T was composed of area S2 (13 × 24 cm), and the arms of the T were composed of areas A1 and A2 (11.5 × 20.5 cm). Areas P1 and P2 were the connecting passageways from the respective arm (area A1 or A2) to the start compartment (area S1). Mice were subjected to a forced alternation protocol for 3 days (one session consisting of 10 trials per day; cut-off time, 50 min). Each trial had first and second runs. On the sample run, the mouse was forced to choose one of the arms of the T (area A1 or A2). After the mouse stayed more than 10 s, the door that separated the arm (area A1 or A2) and the connecting passageway was opened, and the mouse could return to the starting compartment (area S1) via the connecting passageway. The mouse was then given a 3 s delay in area S1, followed by a free choice between both T arms. The correct response was choosing the other arm that had not been chosen on the first trial of the pair. The location of the sample arm (left or right) was varied pseudo-randomly across trials using the Gellermann schedule so that mice received equal numbers of left and right presentations. A variety of fixed extra-maze clues surrounded the apparatus. On days 4-6, a delay (3, 10, 30, or 60 s) was applied after the sample trial. Data acquisition, control of sliding doors, and data analysis were performed by ImageTM software.

**Light/dark transition test**

A light/dark transition test was conducted as previously described (Takao and Miyakawa, 2006). The apparatus consisted of a cage (21 × 42 × 25 cm) divided into 2 sections of equal size by a partition with a door (O’HARA & Co., LTD.). One compartment was brightly illuminated (390 lx), whereas the other compartment was dark (2 lx). Mice were placed into the dark compartment and allowed to move freely between the 2 chambers with the door open for 10 min. The total number of transitions, latency to first enter the lit compartment, distance traveled, and time spent in each compartment were recorded by ImageLD software.

**Elevated plus maze test**

An apparatus consisting of 2 open arms and 2 closed arms was used (O’HARA & Co., LTD.). The open arms (25 × 5 cm) were surrounded by raised ledges (3 mm thick and 3 mm high) to keep mice from falling off the arms. The closed arms were the same size with 15 cm high transparent walls (Komada et al., 2008). This apparatus was made of white plastic. It was elevated 55 cm above the floor. Each mouse was placed in the central square area of the maze (5 × 5 cm) facing one of the closed arms. Mouse behavior was recorded during a 10 min test period. The number of entries into arms, the percentage of entries into and time spent on open arms (%), and distance traveled (cm) were calculated. Data acquisition and analysis were performed automatically using ImageEP software.

**Porsolt forced swim test**

A transparent plastic cylinder (20 cm height × 10 cm diameter) filled with water (21-23°C) up to a height of 7.5 cm was used (Miyakawa et al., 2001). The mouse was placed into the cylinder, and the immobility (%) and distance traveled (cm) were recorded over a 10 min test period on Day 1 and Day 2. Data acquisition and analysis were performed automatically using ImagePS software.

**Tail suspension test**

The tail suspension test was performed for a 10 min test session. Mice were suspended 30 cm above the floor of a white plastic chamber (44 × 49 × 32 cm, inside dimensions; O’HARA & Co., LTD.) in a visually isolated area by adhesive tape placed ~1 cm from the tip of the tail, and the behavior was recorded over a 10 min test period. Images were captured at 2 frames per second. Similar to the Porsolt forced swim test, immobility (%) was judged by the application program according to a certain threshold. Immobility lasting for less than 2 s was not included in the analysis. Data acquisition and analysis were performed automatically using ImageTS software.

**Social interaction test in a novel environment**

The social interaction test was performed to measure social behavior in a novel environment. A pair of age- and weight‐matched mice that had been housed in different cages were placed in a white plastic box (40 × 40 × 30 cm; O’HARA & Co., LTD.) and allowed to explore freely for 10 min. The mice were recorded by a video camera placed above the box. Images were captured at three frames per second and transferred to a computer. The total duration of contacts (s), total number of contacts, total duration of active contacts (s), mean duration per contact (s), and total distance traveled (cm) were automatically calculated by ImageSI software. The "active contact" was measured when the two mice contacted each other and one or both mice moved with a velocity of at least 10 cm/s.

**Sociability and social novelty preference test**

The sociability and social novelty preference test were conducted to assess sociability and social novelty preference. The testing apparatus consisted of a rectangular three‐chambered box and a lid with an video camera (O’HARA & Co., LTD.). The dividing walls of the chamber were made of transparent plastic, with small square openings (5 × 3 cm) allowing access to each chamber (20 × 40 × 47 cm). A small round wire cage (9 cm in diameter, 11 cm in height, with vertical bars 0.5 cm apart) was located in the corner of the left and right chambers. The test mice were first placed in the middle chamber and allowed to explore the entire test chamber for 10 min. Immediately after the 10‐min session, the test mice were placed in a clean holding cage, and a male C57BL/6J mouse (stranger 1) with no prior contact with the test mice was enclosed in one of the wire cages. Next, the test mice were placed in the middle chamber and allowed to explore for 10 min (sociability test). After the sociability session, the test mice were returned to the holding cage, and a second unfamiliar mouse (stranger 2) was enclosed in the wire cage on the opposite side. The test mice were again placed in the middle chamber and had a choice between the first, already investigated unfamiliar mouse and the novel unfamiliar mouse for 10 min (social novelty preference test). The time spent in each chamber and the time spent around each cage were automatically measured from images using ImageCSI software.

**Social interaction test in a home cage**

The home-cage social interaction test was performed using the social interaction monitoring system that consisted of a cage and a cage top with an infrared video camera (25.9 × 15.9 × 23.5 cm, inside dimensions; O’HARA & Co., LTD.). Two mice of the same group that had been housed separately were placed together in the cage. Video images were captured at a rate of 1 frame/s. Social interaction was evaluated by counting the number of animals detected in each image (when the mice were not in contact with each other, they were counted as two particles; when the mice were in contact with each other, they were counted as one particle). The activity level of the mice was also measured by quantifying the number of pixels that changed between each pair of successive frames. Mean number of animals and total activity level in each 1-min bin were calculated for 1 week using the ImageHA software.

**EEG analysis**

EEG and electromyogram (EMG) recordings were performed with *Atp2a2* control and hetero cKO mice (male, 10 months old). Stainless steel screws (1.1 mm in diameter) that served as EEG electrodes were implanted over the somatosensory cortex (1.5 mm lateral to midline, 1.0 mm posterior to bregma) and the hippocampus (2.0 mm lateral to midline, 2.5 mm posterior to bregma) under 1.5% halothane anesthesia with N_2_O:O_2_ (3:2) ventilation 1 week before recording. A reference electrode was implanted on the cerebellum (at midline, 2.0 mm posterior to lambda). EMG electrodes were placed in the cervical region of the trapezius muscle.

**References**

Komada M, Takao K, Miyakawa T (2008) Elevated Plus Maze for Mice. J Vis Exp:108.

Miyakawa T, Yamada M, Duttaroy A, Wess J (2001) Hyperactivity and intact hippocampus-dependent learning in mice lacking the M1 muscarinic acetylcholine receptor. J Neurosci 21:5239–5250.

Takao K, Miyakawa T (2006) Light/dark Transition Test for Mice. J Vis Exp:104.

**Supplementary Figure legends**

**Figure S1 (Related to Figure 4).
Behavioral tests for sensorimotor functions and learning and memory.**

(A) Rotarod test (n = 22 mice per genotype). Latency to fall from the rod. (B) Hot plate test (n = 22 mice per genotype). Latency to withdraw the paw from the hotplate. (C, D) Acoustic startle response/prepulse inhibition tests. Acoustic startle response to 110 dB and 120 dB stimuli (C). Prepulse inhibition of the acoustic startle response with 74 dB and 78 dB prepulse sounds (D). (E-H) Barnes maze test (n = 21 mice per genotype). Latency (E) and number of errors (F) to the visit to the correct hole in the training period (trials 1-18). Time spent around each hole in probe tests performed at 24 h (G) and 30 days (H) after the last training trial. (I, J) T-maze spontaneous alternation test (n = 22 mice per genotype). Percentage of correct responses during the first three sessions with a 3-s delay between trials (I) and during the following three sessions with various delay times (J). Error bars, mean ± SEM.

**Figure S2 (Related to Figure 4).
Behavioral tests for anxiety-like and depression-like behaviors.**

(A-D) Light/dark transition test (n = 22 mice per genotype). The distance traveled in the light/dark compartments (A), time spent in the light compartment (B), number of light/dark transitions (C), and latency to enter the light compartment (D). (E-H) Elevated plus maze test (n = 22 mice per genotype). The number of arm entries (E), percentage of entries into open arms (F), distance traveled (G), and percentage of time spent in open arms (H). (I, J) Porsolt forced swim test (n = 22 mice per genotype). Percentage of immobility time (I) and distance traveled (J) on day 1 and day 2. (K) Tail suspension test (n = 21 mice per genotype). Percentage of immobility time. Error bars, mean ±SEM.

**Figure S3 (Related to Figure 4). Behavioral tests for social interaction.**

(A-E) Social interaction test in a novel environment (n = 11 pairs per genotype). Total duration of contact (A), number of contacts (B), total duration of active contact (C), and mean duration per contact (D), and total distance traveled (E). (F-I) Sociability and social novelty preference test (n = 22 mice per genotype). Time spent in chamber in the sociability test (F). Time spent around cage in the sociability test (G). Time spent in chamber in the social novelty preference test (H). Time spent around cage in the social novelty preference test (I). (J, K) Social interaction test in a home cage (n = 10 pairs, control; n = 9 pairs, hetero cKO). Error bars, mean ± SEM.

**Figure S4 (Related to Figure 4). EEG and sleep/wake cycles.**

(A) Representative EEG patterns recorded in the cortex and hippocampus. (B) Sleep/wake cycles (n = 3 mice per genotype). W, Time awake; NREM, Time in non-REM sleep; REM, Time in REM sleep, Light, light period; Dark, dark period. Error bars, mean ± SEM.

**Figure S5 (Related to Figure 5). Wheel running activity measurements.**

(A) Representative actograms for the control and hetero cKO mice. On the days 41, 55, and 69; actograms around the time of cage changing are marked with orange rectangles. (B) Magnified images of wheel running activity after cage changing (for 90 min; on the day 41). Arrows, time of cage changing. Y axis, wheel running amount. (C) Amount of the wheel running activity for 90 min after cage changing (n = 15 mice per genotype). *, p < 0.05; **, p < 0.001; Mann-Whitney U-test. Error bars, mean ± SEM.
