# Supplementary material for: Brain-specific heterozygous loss-of-function of ATP2A2, endoplasmic reticulum Ca2+ pump responsible for Darier’s disease, causes behavioral abnormalities and a hyper-dopaminergic state
Source: Hum Mol Genet. 2021 Jun 8;30(18):1762–72. doi: 10.1093/hmg/ddab137 (PMC8411987; doi:10.1093/hmg/ddab137)
Supplement: ATP2A2_Fig_S3_ddab137 [file atp2a2_fig_s3_ddab137.pdf]

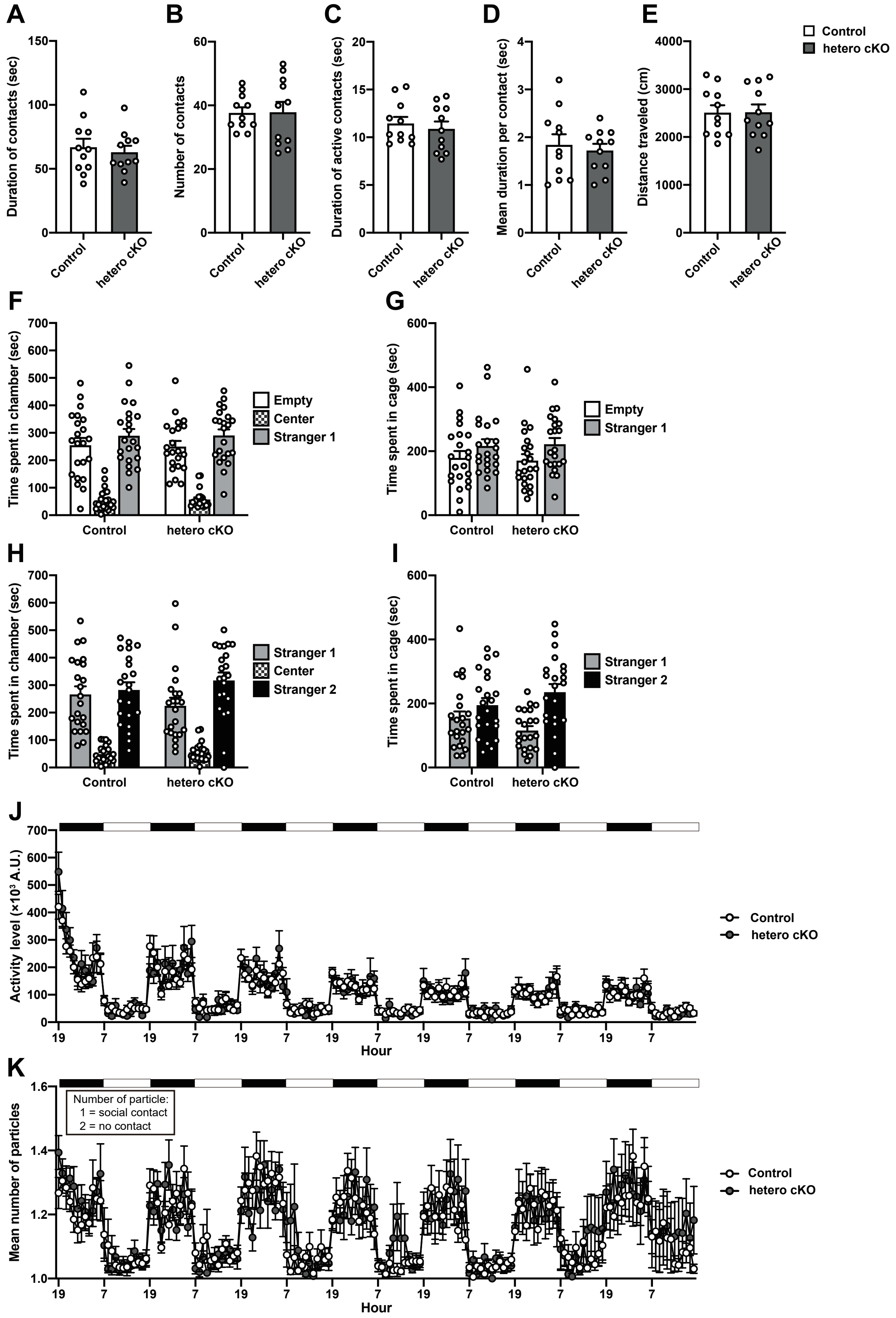

A
B
C
D
E
Control
hetero cKO
Duration of contacts (sec)
Number of contacts
Duration of active contacts (sec)
Mean duration per contact (sec)
Distance traveled (cm)
F
G
Time spent in chamber (sec)
Time spent in cage (sec)
Empty
Center
Stranger 1
H
I
Stranger 2
J
Activity level (×10³ A.U.)
Hour
K
Mean number of particles
Number of particle:
1 = social contact
2 = no contact


# Figure S3
